# Subglottic hemangiome in childhood

**DOI:** 10.1590/S1808-86942012000100025

**Published:** 2015-10-20

**Authors:** Tiago Neves Veras, Rafaela Campos Benvenutti, Gilberto Hornburg, Adrian Mauricio Stockler Schner

**Affiliations:** a MD. MSc.; b MD. Pediatric Pneumologist; c MD. Radiologist; d MD. Chest Surgeon - Hospital Municipal São José, Joinville

**Keywords:** hemangioma, infant, respiratory sounds

## INTRODUCTION

Subglottic hemangioma is one of the most common airway vascular neoplasms in children. It usually presents with inspiratory or biphasic stridor, increased by feeding and upper airway infections^1^. During its proliferative stage, usually before 12 months of age, it can be fatal if diagnosis and treatment are not started quickly^2^.

Surgical treatment is based on tracheostomy, surgical excision of the lesion, local injection of steroids and laser^2^. Recently, several authors have proposed a clinical management with propranolol, yielding good results^3^. The goal of the present study was to describe an infant with inspiratory stridor developed after hospitalization for acute viral bronchiolitis, with a final diagnosis of subglottic hemangioma and indication of clinical treatment.

## CASE REPORT

An eight month-old female, with a history of stridor perceived by the mother, especially during sleep. She was hospitalized at four months of age because of an acute viral bronchiolitis (without findings of respiratory viruses). She required supplemental oxygen for three days and symptomatic medication. She was breastfed only until the fourth month without significant perinatal history. She had normal PKU test, proper weight and height gains, and had been properly vaccinated. The mother reported stridor improvements with oral steroids.

**Figure 1:**
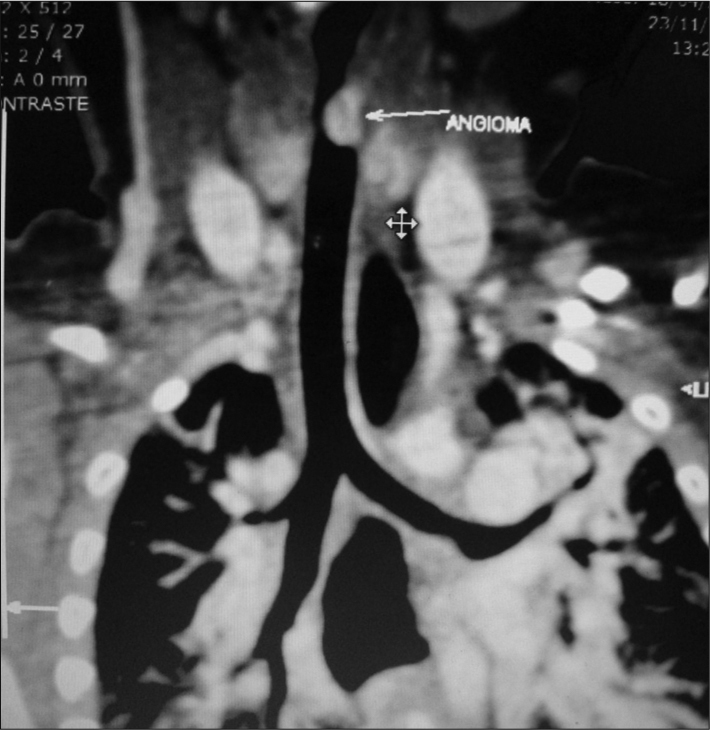
Chest and neck CT scan showing an angioma

Upon physical examination, the infant was eutrophic, with mild inspiratory stridor; and rare transmission snoring upon pulmonary auscultation. A high resolution chest CT scan associated with virtual bronchoscopy showed a vascular lesion in the subglottis, blocking about 90% of the airway (Figure 1). The proposed treatment was propranolol (2mg/kg/day) associated with prednisolone (1mg/kg).

## DISCUSSION

Stridor in infants may be translated into a wide range of conditions, some of them potentially life-threatening, such as subglottic hemangioma with airway obstruction. As congenital causes, we list: laryngomalacia, subglottic stenosis, vocal cord paralysis, laryngocele and laryngeal membranes. The main acquired causes are foreign body aspiration, hemangiomas, recurrent papillomatosis and croup^4^. Hemangiomas may spontaneously regress later on, by 5 years of age.^5^

Until recently, the best treatment for this type of condition was surgery, with reports of up to 94.5% of success in some series. However, because of the rich vascularization of the lesion and its anatomical location, the risks inherent to the procedure should not be disregarded. Thus, the use of clinical treatment represents an important advance for this condition.

Monotherapy with oral steroids may fail in up to 75% of the patients, and it has its side effets^6^. Beta-blockers, such as propranolol, may represent an inexpensive alternative, which is also non-invasive and well-tolerated by the patient^4^; not forgetting the paramount need for a cardiological assessment of the patient (echocardiogram and electrocardiogram) before treatment onset. The mechanism of action involved is related to the inhibition of mitogenic factors stimulating apoptosis in capillary endothelial cells. The aforementioned patient has been asymptomatic after one month of therapy, and will soon be off the steroids.

## FINAL REMARKS

Treatment with propranolol is inexpensive, noninvasive and well tolerated, it may prevent the patient from being submitted to surgery or event tracheostomy because of disease progress. Such medication can cause bradycardia, decreased blood pressure and hypoglycemia. The authors' recommendation is that once the subglottic hemangioma is diagnosed in children, propranolol is a valuable tool for treatment, pending a paramount cardiac evaluation prior to the start of medication.
